# Accurate Diagnosis of Lower Respiratory Infections Using Host Response and Respiratory Microbiome from a Single Metatranscriptome Test of Bronchoalveolar Lavage Fluid

**DOI:** 10.1002/advs.202405087

**Published:** 2024-12-18

**Authors:** Xiaohui Zou, Mengwei Yan, Yeming Wang, Yawen Ni, Jiankang Zhao, Binghuai Lu, Bo Liu, Bin Cao

**Affiliations:** ^1^ National Center for Respiratory Medicine State Key Laboratory of Respiratory Health and Multimorbidity National Clinical Research Center for Respiratory Diseases Institute of Respiratory Medicine Chinese Academy of Medical Sciences Department of Pulmonary and Critical Care Medicine Center of Respiratory Medicine China‐Japan Friendship Hospital Beijing 100029 China; ^2^ Department of Clinical Microbiology Pulmonary and Critical Care Medicine Zibo City Key Laboratory of Respiratory Infection and Clinical Microbiology Zibo City Engineering Technology Research Center of Etiology Molecular Diagnosis Zibo Municipal Hospital Zibo 255400 China; ^3^ Weifang People's Hospital Shandong Second Medical University Weifang Shandong Province 261041 China; ^4^ Department of Pulmonary and Critical Care Medicine Shandong Institute of Respiratory Diseases The First Affiliated Hospital of Shandong First Medical University Shandong Provincial Qianfoshan Hospital Shandong University Jinan 250014 China

**Keywords:** host response, lower respiratory tract infection, respiratory microbiome

## Abstract

Lower respiratory tract infections (LRTIs) diagnosis is challenging because noninfectious diseases mimic its clinical features. The altered host response and respiratory microbiome following LRTIs have the potential to differentiate LRTIs from noninfectious respiratory diseases (non‐LRTIs). Patients suspected of having LRTIs are retrospectively enrolled and a clinical metatranscriptome test is performed on bronchoalveolar lavage fluid (BALF). Transcriptomic and metagenomic analysis profiled the host response and respiratory microbiome in patients with confirmed LRTI (*n* = 126) or non‐LRTIs (*n* = 75). Patients with evidenced LRTIs exhibited enhanced pathways on chemokine and cytokine response, neutrophile recruitment and activation, along with specific gene modules linked to LRTIs status and key blood markers. Moreover, LRTIs patients exhibited reduced diversity and evenness in the lower respiratory microbiome, likely driven by an increased abundance of bacterial pathogens. Host marker genes are selected, and classifiers are developed to distinguish patients with LRTIs, non‐LRTIs, and indeterminate status, achieving an area under the receiver operating characteristic curve of 0.80 to 0.86 and validated in a subsequently enrolled cohort. Incorporating respiratory microbiome features further enhanced the classifier's performance. In summary, a single metatranscriptome test of BALF proved detailed profiles of host response and respiratory microbiome, enabling accurate LRTIs diagnosis.

## Graphical abstract

This study addresses the diagnostic challenges of lower respiratory tract infections (LRTIs) by leveraging metatranscriptomic analysis of bronchoalveolar lavage fluid. It efficiently detects a broad range of pathogens, while also identifying distinct host immune responses and microbial signatures associated with LRTIs. Utilizing these insights, the study develops a high‐accuracy machine learning classifier and enhances LRTIs diagnostic precision.

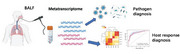

## 1 Introduction

Lower respiratory tract infection (LRTI) causes more deaths each year than any other type of respiratory disease.^[^ ^1^ ^]^ A wide spectrum of microorganisms could cause LRTI, including bacteria, virus, fungi, and mycobacterium, making accurate diagnosis of causing agents challenging. Moreover, opportunistic pathogens and normal resident bacteria could also lead LRTI under specific scenarios, such as in immune compromised patients.^[^ ^2^, ^3^ ^]^ Currently, LRTI pathogens detection mainly relies on culture, urine/sputum antigen testing, molecular diagnostic assays in the clinical microbiology laboratory. Molecular detection assays provide a decent turnaround time (generally 2–6 h) and express high specificity and sensitivity for the detection of common pathogens.^[^ ^4^ ^]^ However, these conventional microbiological tests target only one or a limited panel of pathogens at a time or require that a microorganism be successfully cultured from clinical samples; due to these limitations of current tools, possible bacterial pathogens are only detected in 20–50% of patients and a distinction between colonization and infection is difficult.^[^ ^5^, ^6^ ^]^

Clinical metagenomic/ metatranscriptomic next‐generation sequencing (mNGS) is a promising technique to improve the clinical capacity on LRTI diagnosis;^[^ ^7^, ^8^ ^]^ mNGS assasy applied parallel deep sequencing of all the genetic material (DNA and RNA) and provide a comprehensive view of all the microbes in the sample, enabling full‐spectrum pathogen detection.^[^ ^9^ ^]^ Due to the broad spectrum of pathogens causing LRTIs and the limited identification rates of current diagnosis tools, mNGS is increasingly applied on LRTIs diagnosis and exhibited clinical value on difficult‐to‐diagnose cases and emerging infectious diseases caused by novel microbes.^[^ ^10^, ^11^ ^]^ When mNGS test was applied to LRTI diagnosis, brochoalveolar lavage fluid (BALF) was the primary sample type as it was collected from the lower respiratory tract,^[^ ^12^ ^]^ precisely where the infection occurs. However, the commensal microbiota and opportunistic pathogens presenting in LRTI specimens complicated the interpretation of mNGS report. Specifically, the clinicians face challenges in determining whether the patients are afflicted with LRTIs or are experiencing non‐infectious conditions that mimic LRTI symptoms.

Host response to infection has emerged as a promising tool for accurate LRTI diagnosis in critically ill adults and children.^[^ ^13^ ^]^ Host response analysis has shown that certain immunological pathways are upregulated following pathogen infection, particularly those involving cytokine production, chemokine signaling, and inflammatory responses.^[^ ^14^ ^]^ These pathways play critical roles in the body's defense mechanism against infections, enhancing the ability of immune cells to respond to and clear pathogens.^[^ ^15^ ^]^ This upregulation serves as a biomarker for distinguishing between infectious and non‐infectious conditions, thereby improving the diagnostic precision of LRTI.^[^ ^16^ ^]^ On the other hand, changes in the lower respiratory tract microbiome following LRTIs can also serve as crucial diagnostic markers.^[^ ^17^ ^]^ Typically, LRTIs are associated with a marked decrease in microbial diversity and a shift in the composition of the microbiome.^[^ ^18^ ^]^ This often includes an increase in the relative abundance of pathogenic bacteria and a decrease in commensal species,^[^ ^19^ ^]^ which can disrupt the normal microbial balance. Furthermore, the presence of specific pathogens or a characteristic microbial profile in the bronchoalveolar lavage fluid (BALF) can be indicative of LRTIs,^[^ ^20^ ^]^ aiding in the differentiation from non‐infectious respiratory conditions. This microbial signature, combined with host immune response data, enhances the diagnostic accuracy and helps in the timely and effective management of the disease.

Here, we characterized the host response and respiratory microbiome using a single metatranscriptomic sequencing of BALF sample from a retrospective cohort consisting of 201 patients with LRTIs or non‐infectious mimic diseases. We observed distinct host pathway activation and respiratory microbiome features between patients with LRTIs and non‐LRTIs. We then developed a LRTI diagnosis classifier that integrated both host and microbiome features and demonstrated good performance in differentiating patients with LRTIs from those with noninfectious mimics.

## 2 Results

### 2.1 Clinical Features of Study Cohort

**Figure 1:**
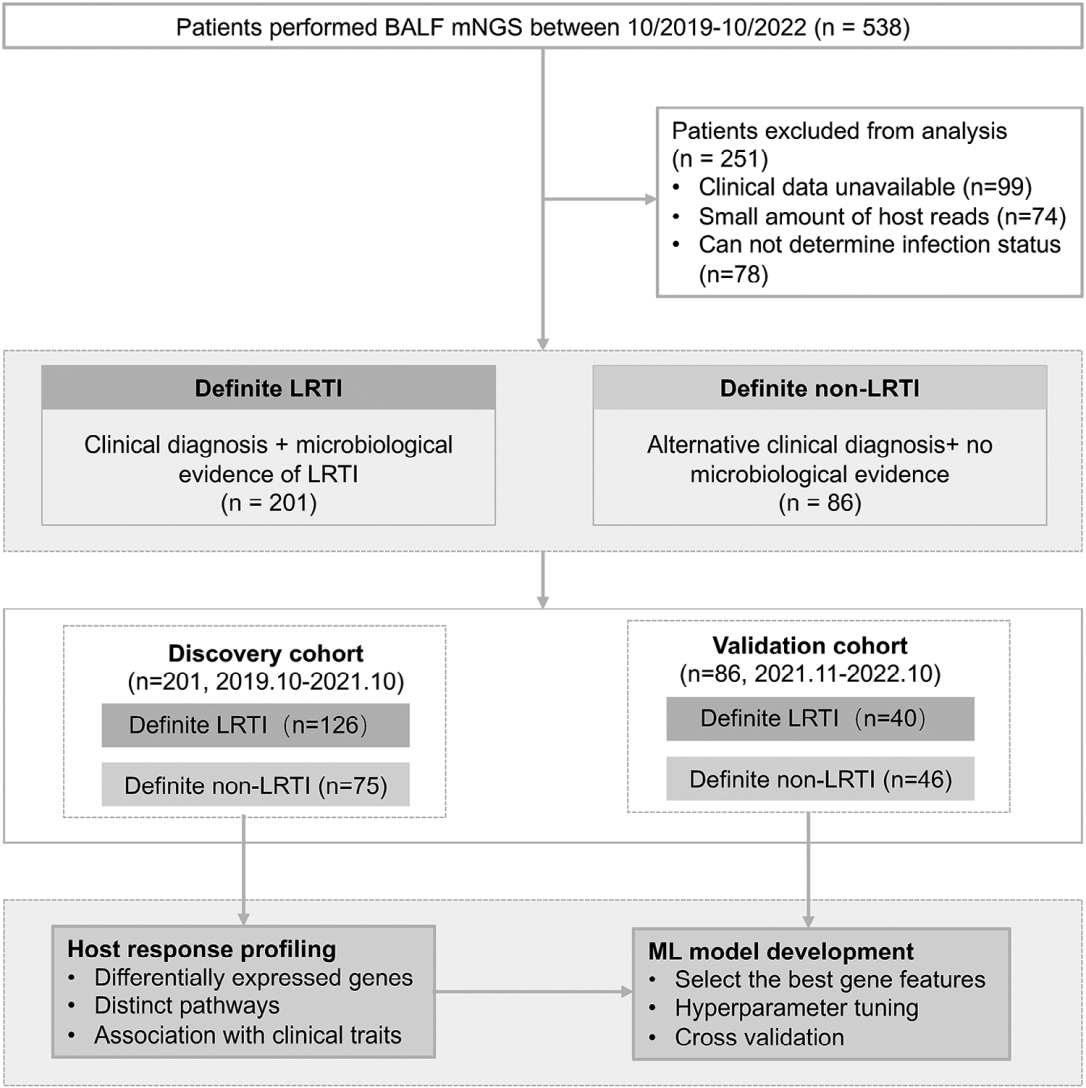
Study flow chart. Patients suspected to have LRTI and with a BALF mNGS test were clinically adjudicated into definite LRTI and non‐LRTI status. Patients hospitalized through Oct 2019 to Oct 2021 were served as discovered cohort to profile host response and classifier establishment. The patients admitted between Nov 2021 to Oct 2022 constituted the validation cohort and their host response were used to validate the classifier's performance in novel dataset.

A total of 538 patients who underwent bronchoscopy and BALF mNGS were assessed according to clinical data in the EMR system; 251 patients were removed for incomplete clinical data, undetermined infection status, and insufficient host reads (< 3 ×10^5^) (**Figure** **1**). Patients were categorized of two groups based on the final clinical diagnosis and clinical microbiology evidence (Experimental Section). We finally involved 201 patients in the discovery cohort and 86 in the validation cohort, with 74 (36.8%) and 30 (34.9%) were female, respectively (**Table** **1**). The median age in both cohorts was close, with 57 (28.4%) and 29 (33.7%) being immunocompromised, respectively; of these, 9 and 1 were hematology patients, respectively (Table S2, Supporting Information). 26.4% and 29.1% of patients were admitted to intensive care unit (ICU) and 14 (7.0%) and 8 (9.3%) patients were deceased finally in discovery and validation cohort, respectively. Bacteria were the most common pathogens in both cohorts (54.0% versus 35.0%), followed by viruses (18.3%) in the discovery cohort and mixed pathogens (22.5%) in the validation cohort. Antibiotics were used in 54 and 48 patients among the 132 and 68 patients with clear antibiotic usage information in discovery and validation cohort, respectively. Within the discovery cohort, 126 (62.7%) patients were assigned to LRTIs and 75 (37.3%) to non‐LRTI group, which were used to build a ML classifier based on the DEGs in the BALF; the validation comprising 40 (46.5%) LRTI patients and 46 (53.5%) non‐infection patients were utilized to assess the robustness of the model in handling new dataset.

**Table 1:** Demographic and clinical cohort characteristics.

		Discovery cohort (n = 201)	Validation cohort (n = 86)
Patient characteristics			
	Female,n (%)	74 (36.8%)	30(34.9%)
	Male, n (%)	127 (63.2%)	56 (65.1%)
	Age, median (IQR1, 3)	61(51‐69)	62 (53‐68)
Clinical metrics^#)^			
	Immunocompromised, n (%)	57 (28.4%)	29 (33.7%)
	ICU, n (%)	53 (26.4%)	25 (29.1%)
	Infection, n (%)	126 (62.7%)	40 (46.5%)
	Death, n (%)	14 (7.0%)	8 (9.3%)
Blood count^$)^		Discovery cohort (n = 165)	Validation cohort (n = 80)
	WBC, median (IQR)	7.58 (5.57‐11.70)	4.92 (3.58‐6.95)
	NEU, median (IQR)	5.42 (3.73‐9.69)	7.17 (5.38 – 9.02)
	LYMP, median (IQR)	1.15 (0.68‐1.68)	1.28 (0.79‐1.88)
Pathogens in LRTI^*)^		Discovery cohort (n = 126)	Validation cohort (n = 40)
	Bacteria	68 (54.0%)	14 (35.0%)
	Viral	23 (18.3%)	9 (22.5%)
	Fungi	17 (13.5%)	5 (12.5%)
	Co‐infection	18 (14.3%)	12 (30.0%)
TOP5 pathogens in LRTI			
	CMV	22 (17.5%)	9 (22.5%)
	KPN	18 (14.3%)	4 (10.0%)
	SPN	18 (14.3%)	1(2.5.%)
	PAE	18 (14.3%)	6 (15.5%)
	PCP	16 (12.7%)	6 (15.5%)

P values comparing patients in the two cohorts. Mann–Whitney test was used for all continuous variables. Fisher's exact test was used for all categorical variables.

^#)^ Clinical metrics include patients’ immune status (Immunocompromised), patients who required admission to the intensive care unit (ICU) during their treatment, patients who were confirmed to have lower respiratory tract infections (Infection), and patients who died during the hospitalization (Death).

^$)^ Blood count information was available for 165 patients in the discovery cohort and 80 patients in the validation cohort, within one day of the BALF sampling. WBC: White Blood Cell Count, NEU: Neutrophils count, LYMP: Lymphocytes count.

*) Pathogens in LRTIs were summarized as bacterial, viral, fungi, and co‐infections involving different types of pathogens. The top 5 pathogens most frequently detected in the discovery cohort, including cases of co‐infections, was listed below. CMV: Cytomegalovirus, KPN: Klebsiella pneumoniae, SPN: Streptococcus pneumoniae, PAE: Pseudomonas aeruginosa, PCP: Pneumocystis jirovecii.

### 2.2 Distinct Host Response Revealed by BALF RNA‐seq Data Between LRTIs and Non‐LRTIs

**Figure 2:**
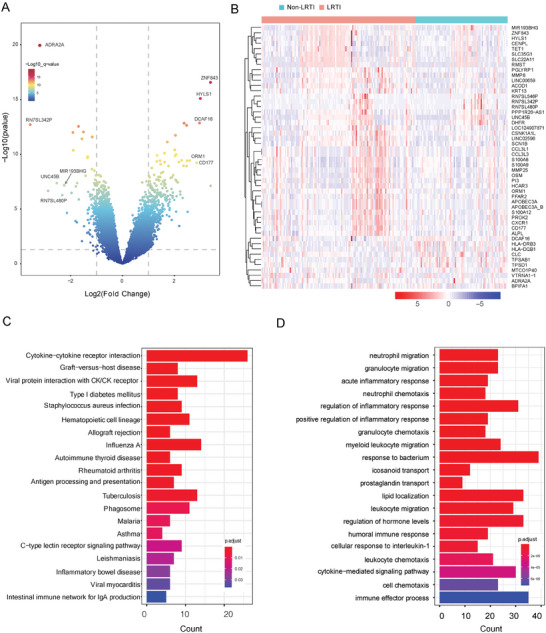
Distinct host response profiled from mNGS host data between patients with LRTIs and non‐LRTIs. A) The differentially expressed genes (DEGs) between LRTI and non‐LRTI patients. The top 5 genes with the highest fold change upregulated in each group (left: non‐LRTI; right: LRTI) were labeled. B) Heat map of expression level of the top 50 DEGs in the two groups. Color range representing the normalized gene expression values are attached below. C) The top 20 KEGG pathways that are enriched with DEGs. D) The top 20 GO terms that are enriched with DEGs. The enriched pathways and GO terms are predominantly related to the innate immune response against viral and bacterial infections.

We performed transcriptome analysis on the RNA‐seq data from BALF mNGS test to assess the distinct host response between patients with LRTIs and non‐infectious illness. The 201 BALF samples in the discovery cohort produced a median of 9.1 × 10^6^ (IQR1‐IQR3: 5.5×10^6^–12.7×10^6^) reads per sample, which identified 766 DEGs between LRTIs and non‐LRTIs (**Figure** **2**A). RN7SL342P and ZNF483 were the top down‐ and up‐regulated DEGs with the most significant fold change in the LRTI patients (Figure 2A). We visualized the top 50 DEGs and observed that most upregulated genes are involved in the innate immune response against infection (Figure 2B). Notably, LRTIs patients expressed higher level of chemokine ligand and receptors genes, including CCL3L1, CCL3L3, CXCR1, which play a role to recruit immune cells to the lower respiratory tract. Another two genes, the S100A8 and S100A9, which stimulated leukocyte recruitment and induce cytokine secretion, also expressed at a higher level in the LRTIs patients; meanwhile, several viral‐infection‐related genes also expressed at high level in LRTIs patients, including FFAR2, OSM, and ORM1. We also detected higher level of MMP8 in the LRTIs, which coordinated leukocyte trafficking through cleavage of collagen and chemokine‐binding protein.

The universal up‐regulated proinflammatory genes indicated distinct pathway activation between LRTIs and non‐infection. We then performed KEGG pathway enrichments analysis on the DEGs detected above (Figure 2C). As expected, the top1 and top 3 enriched pathways were related to cytokine response after infection, namely, the “Viral protein interaction with cytokine/receptor” and “Cytokine−cytokine receptor interaction”. Besides cytokine response, the pathways involved in the host response against common respiratory pathogens infection, such as “Staphylococcus aureus infection”, “Influenza A”,“ Tuberculosis”, were enriched in the DEGs. Other pathways involved in innate and adaptive immune response against respiratory infection were also enriched, including “Antigen processing and presentation”, “Phagosome”, and “ C−type lectin receptor signaling pathway”.

The GO enrichment analysis further indicated universal activation of innate immune response against respiratory infection (Figure 2D). Typically, neutrophil migration and chemotaxis were top enriched process, followed by GO terms involved inflammatory response, such as “acute inflammatory response” and “regulation of inflammatory response”. Other GO terms related to myeloid/leukocyte activation, migration, and chemotaxis, were also enriched by these DEGs.

### 2.3 Host Response of LRTIs was Associated with Patients' Clinical Features

**Figure 3:**
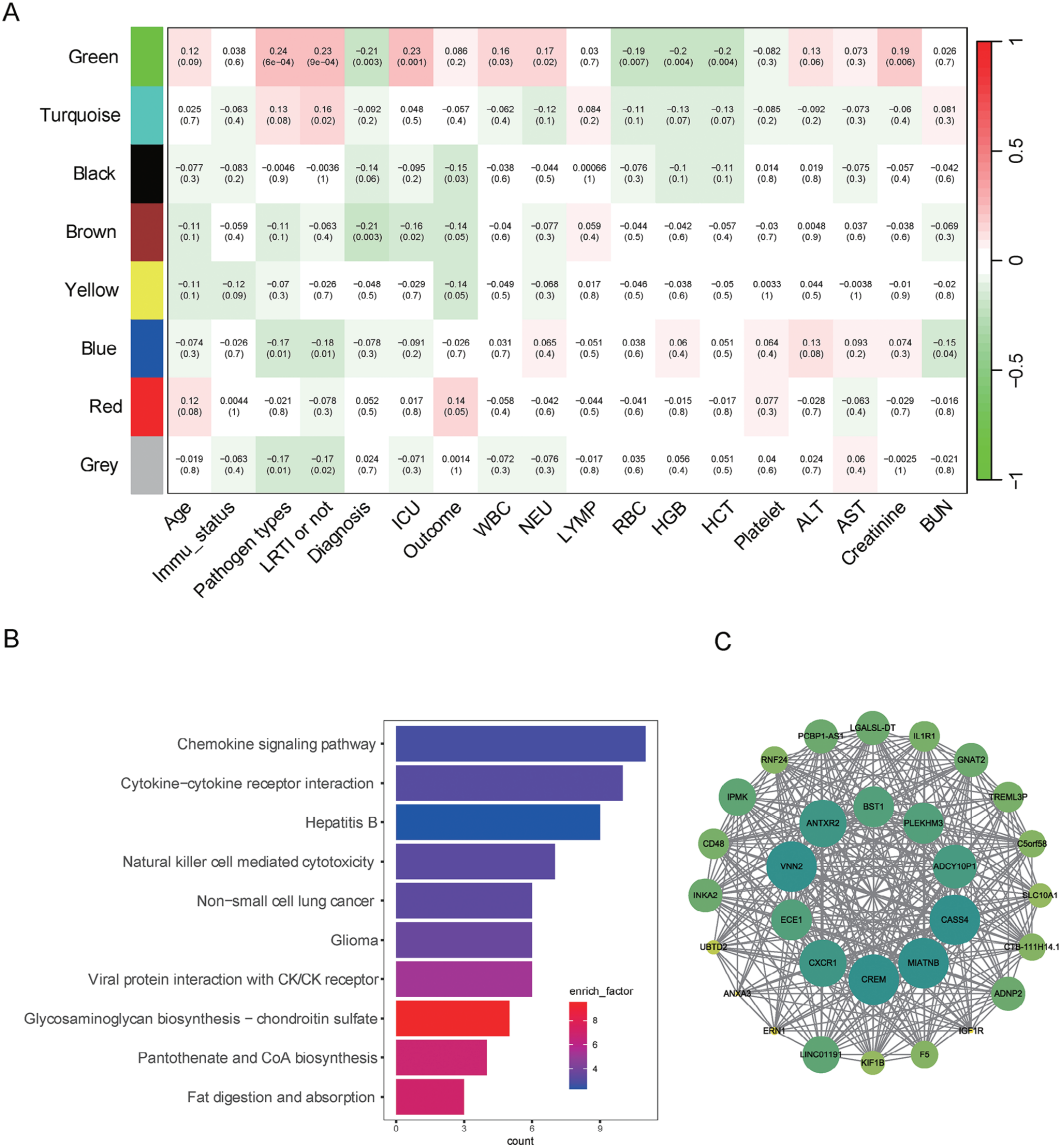
Association between gene module and clinical traits. A) Heatmap of Pearson correlation coefficient between gene module and patients’ clinical traits. Each cell reports the correlation (and p‐value) between module eigengenes (rows) and clinical features. The red and green colors indicate strong positive correlation and strong negative correlation, respectively. LYMP: lymphocyte count; HGB: hemoglobin; HCT: hematocrit; BUN: blood urea nitrogen. B) The enriched KEGG pathways with genes in the green module, mainly involved on chemokine and cytokine response following infection. C) The top 30 genes with highest intra‐modular connectivity are shown in the network, which demonstrated intensive interactions between each other.

The BALF mNGS RNA‐seq data revealed distinct gene expression and pathway activation between the two cohorts. Consequently, we sought to investigate whether the host response derived from mNGS was related to the patients' clinical features. A total of 10 889 genes and 198 patients were included in the WGCNA analysis after the removal of genes with low expression and outlier patients. WGCNA clustered all these genes into 8 gene modules, from which many have significant association with clinical metrics (*p* < 0.05) (**Figure** **3**A). The correlation analysis indicated that the green module was positively correlated with patient's LRTI status, pathogen types, ICU admission, white blood cell counts, and neutrophil counts in the blood. KEGG enrichment analysis revealed that “Chemokine signaling pathway” and “Cytokine−cytokine receptor interaction” pathways were the top2 enriched ones in the green module (Figure 3B). Other pathways in the anti‐infection immune response, including “Natural killer cell mediated cytotoxicity”, “Viral protein interaction with cytokine/cytokine receptor” were also enriched in this module. The top 30 genes with highest intra‐modular connectivity were extracted to build a gene‐related Protein‐Protein Interaction (PPI) network (Figure 3C). The PPI observed that the hub genes with the largest number of associated nodes in the weighted network played a crucial role in the immune cell recruitment and activation after infection, such CXCR1, VNN2, BST1, CREM, IL1R1, and CD48. These results confirmed that genes in the green module involved the host response against infection and associated with patient's clinical features.

### 2.4 Classification of LRTI Status Based on Host Gene Expression Features

The BALF mNGS RNA‐seq data observed a clear signature of infection in the LRTIs group, characterized by activated innate immune pathways against infection (Figure 2). Thus, we first sought to develop a ML classifier to discriminate LRTI patients from its mimic non‐LRTIs, based on the host gene expression features from BALF mNGS. Only DEGs identified by edgeR in the discovery cohort were considered as potential predictors and included in ML models. We used the normalized reads count of each DEGs to establish RF, LR, and SVM model, which achieved 69.2% ± 8.1%, 74.1% ± 6.0%,70.2% ± 6.7% accuracy in the 5× cross validation. We selected LR for features selection and hyper parameter tuning for its better performance on whole DEGs.

**Figure 4:**
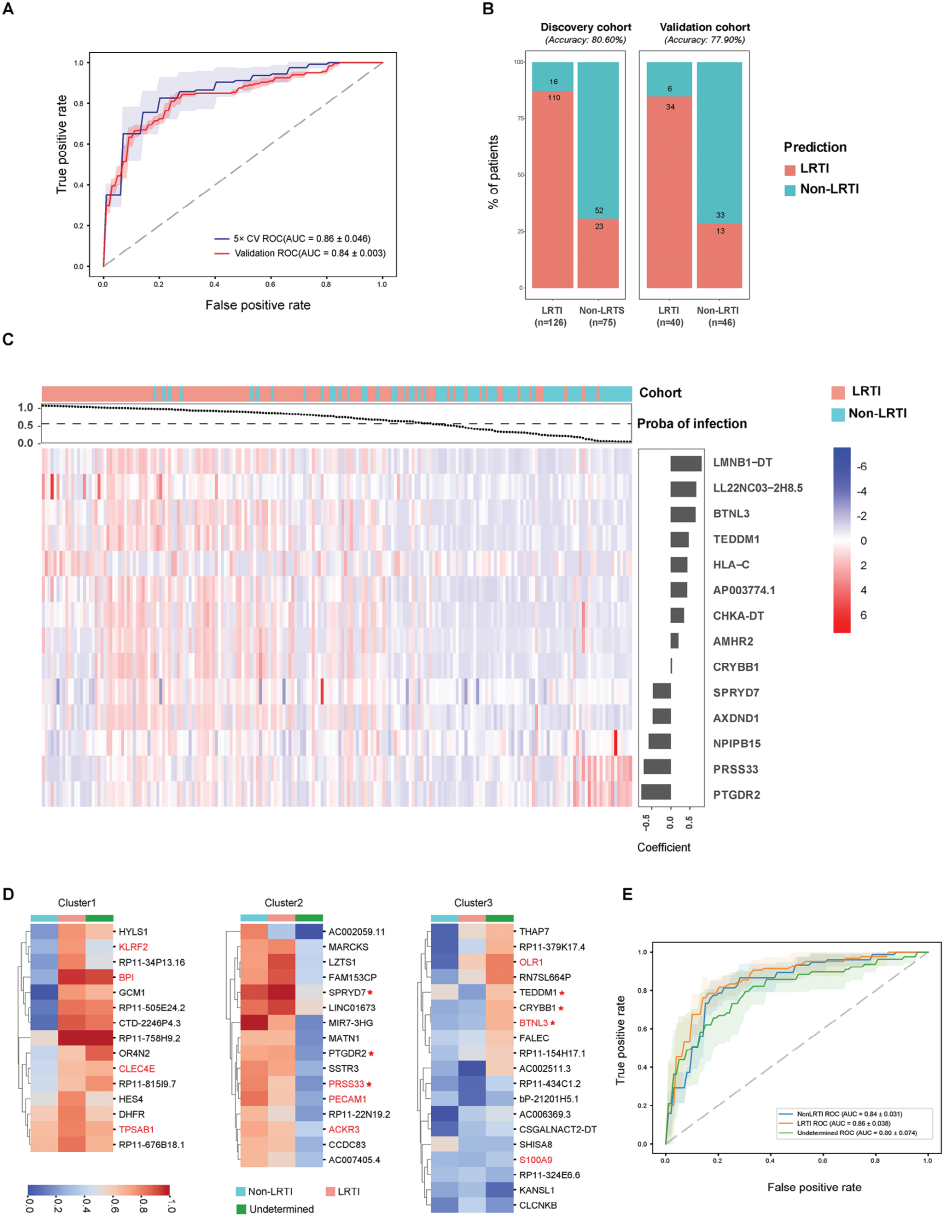
Performance of host gene expression classifier for LRTI diagnosis. A) Receiver operating characteristic (ROC) curve of the host gene expression classifier. The area under curve (AUC) values and s.d. are listed for fivefold cross‐validation in the discovery cohort (blue line: average AUC of each test folds; blue shaded area: ±1s.d.), and in the validation cohort (red line and red area). B) The number and percentage of patients predicted to be LRTI and non‐LRTI by host‐based classifier in discovery and validation cohort. C) The normalized expression levels of the 14 final selected classifier genes across all patients (columns) in the discovery cohort. Patients LRTI status were marked with top color horizontal bar and out‐of‐fold LRTI probability for each patient were attached below. The regression coefficient of each selected gene was denoted by side bar plot. D) Coefficients of the 50 feature genes for each category in the three‐class SVM model, clustered based on coefficients. Immune‐related genes are labeled in red, with genes overlapping with the two‐class model marked with a star (E) ROC and AUC values (±1s.d.) for each category of the three‐class SVM model based on fivefold cross‐validation.

We tested the number of features to select between 5 to 20 and achieved the best accuracy of 80.5% ± 7.9% when selecting a final set of 14 genes using the RFE method. The LR model accuracy slightly increased to 80.6% after parameter tuning and achieved average AUC of 0.86 ± 0.046 over fivefold cross‐validation within the discovery cohort (**Figure** **4**A). We then tested the model robustness in a validation cohort enrolled in subsequent period and the model achieved 77.9% accuracy (Figure 4B).

The 14 selected genes showed distinct expression between LRTIs and non‐LRTIs (Figure 4C). The top 3 genes with positive regression coefficients were LMNB1−DT, LL22NC03−2H8.5, and BTNL3, while PTGDR2, PRSS33, and NPIPB15 were the top 3 genes negatively correlated with LRTIs. The majority of patients with LRTIs achieved a high predicted probability value and most patients with non‐LRTIs have low value, indicting the high accuracy. Moreover, the model also made a precise prediction in patients with bacteria, viral, fungi, and co‐infections of distinct pathogens (Figure S1, Supporting Information).

Patients who are difficult to diagnose with LRTI are common in clinical scenarios. Thus, we included these patients into the discovery cohort, alongside those with a definite diagnosis, to establish a three‐category model that classify patients into three categories: LRTI, non‐LRTI, and undetermined LRTI. The feature selection process selected out 50 DEGs to train a SVM model considering balance of model complexity and performance using the RFE method. Many of the selected genes were immune‐related and play a role in the host response against infection, such as BPI, PRSS33, S100A9, CLEC4E, and so on. Moreover, these genes formed three distinct clusters based on their coefficients with each cohort, reflecting their relative importance in determining the respective category (Figure 4C; Figure S2, Supporting Information). This three‐class model demonstrated an accuracy of 72% ± 9.3% in the 5× cross‐validation dataset containing 126 LRTI, 75 non‐LRTI, and 78 undetermined LRTI patients. The patients with undetermined LRTI status showed the lowest AUC when evaluating the ROC for each individual category (Figure 4E).

### 2.5 LRTI Microbiome Features and Classification of LRTI Status Based on the Integrated Model

**Figure 5:**
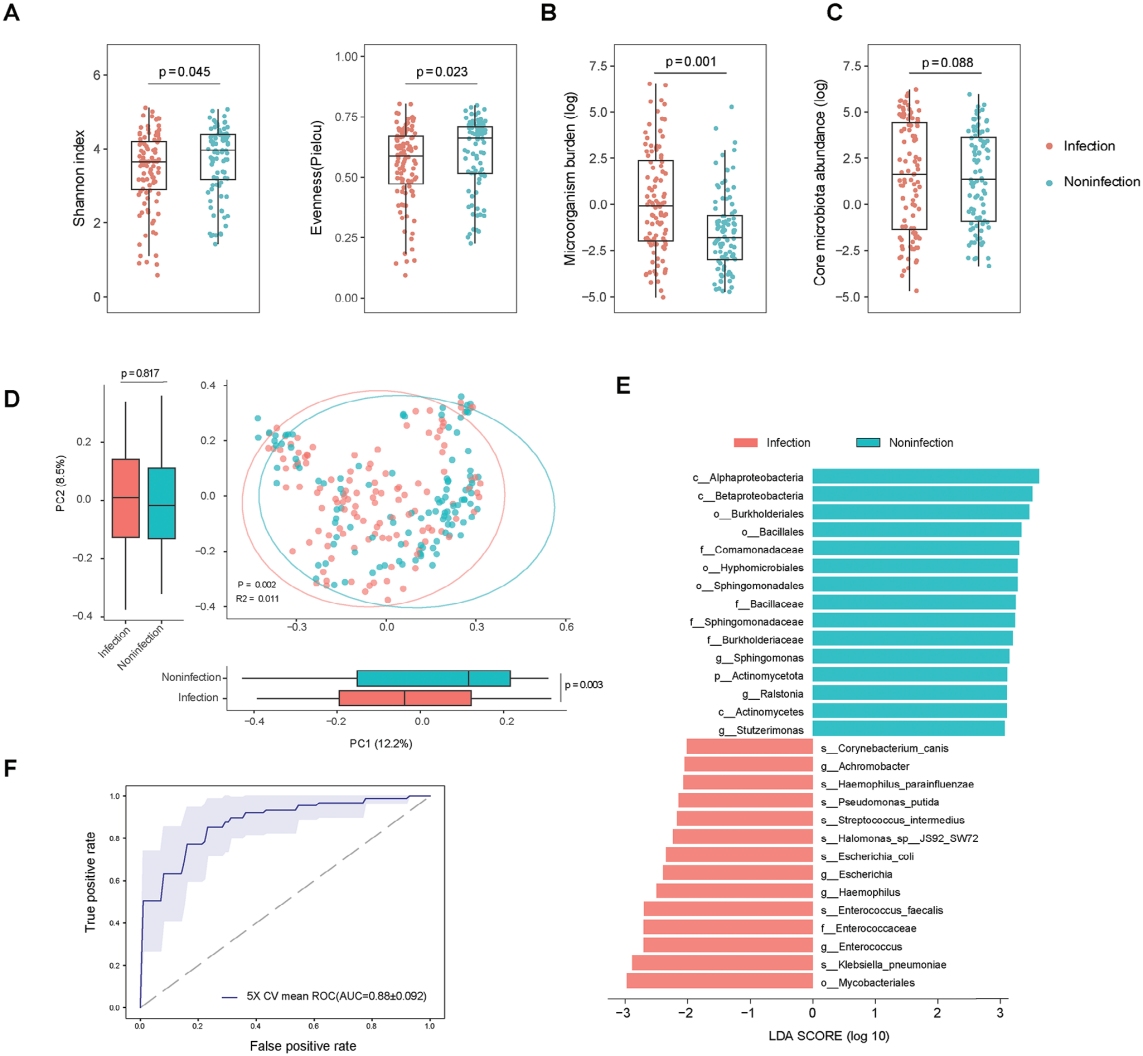
Respiratory microbiome in LRTI patients and integration model of host response and microbiome A) Comparison of alpha diversity (Shannon index), evenness (Pielou index), and microorganism burden (log‐transformed ratio of reads from the Kingdoms of bacteria, fungi, and viruses to total reads) between patients with LRTIs (Infection) and noninfectious respiratory diseases (Noninfection), with age and antibiotic usage adjusted using general linear model. B) Principal coordinate analysis (PCoA) plots showing the separation of respiratory microbiome profiles between patients with LRTIs and those with noninfectious respiratory diseases based on Bray–Curtis dissimilarity. C) Comparison of the percentage of reads from the pulmonary core microbiota between LRTI and non‐LRTI patients. D) Linear discriminant analysis (LDA) score plot highlighting key bacterial taxa that distinguish between LRTI (Infection) and non‐LRTI (Noninfection) patients. E) Receiver operating characteristic (ROC) curve of the integration classifier which use both host and respiratory microbiome features. blue line: average over 5 random splits; shaded area: ±1 s.d.

A total of 232 samples (154 in discovery and 78 in validation cohort) with metagenomic coverage above 60%, as estimated by Nonpareil^[^ ^21^ ^]^ after host reads removal, were retained for respiratory microbiome analysis. The alpha diversity and evenness were lower in LRTI patients than that in noninfectious disease (**Figure** **5**A). Moreover, the microorganism burden was higher in LRTIs patients, indicated by an increased percentage of reads from microorganisms compared to patients with non‐infectious diseases (Figure 5A). These differences remained significant after adjusting for covariates such as antibiotic usage and age. However, no difference was observed in the percentage of reads from the pulmonary core microbiota between the two groups (Figure 5C).

The BLAF metatranscriptome sequencing also provide detailed respiratory microbiome profile, which in patients with LRTIs were distinct from those non‐infectious diseases using principal coordinate analysis (PCoA) (Figure 5D). Specifically, bacteria species commonly caused CAP, such as *Escherichia_coli* and *Klebsiella_pneumoniae*, and genus containing many CAP bacteria pathogen, such *as Escherichia, Haemophilus*, and *Achromobacte*, was highly enriched in LRTIs patients. Moreover, gut‐associated bacterial taxa, including the family *Enterococcaceae*, genera such as *Escherichia* and *Enterococcus*, and species like *Escherichia coli, Enterococcus faecalis*, and *Klebsiella pneumoniae*, were also enriched in LRTI patients.

Next, we examined whether integrating the microbial features that are distinct between the two cohorts into the host classifiers could improve LRTI diagnosis. Three microbiome features, the alpha diversity (Shannon index), evenness (pielou index), and microorganism burden (percentage of microorganism reads to total reads), were incorporated into the abundance data of 14 selected genes to train a LR model, with hyperparameter tuning as described above. The integrated model achieved a slightly increased AUC of 0.88 ± 0.092 when assessed by fivefold cross‐validation, indicating that integrating microbiome features enhances the performance of the host classifier.

## 3 Discussion

LRTIs are a leading cause of morbidity and mortality worldwide, particularly among vulnerable populations like children, elders, and individuals with weakened immune systems. LRTIs can result in severe illness, hospitalizations, and death in severe cases. Diagnose of LRTIs can be challenging due to the diverse spectrum of pathogens involved, and non‐infectious factors like allergens or pollutants, which lead overlapping symptoms and make it difficult to pinpoint the specific pathogen responsible.^[^ ^22^ ^]^ Metatranscriptome/metagenomic NGS is increasingly applied in the diagnosis of LRTIs due to several advantages.^[^ ^23^ ^]^ It allows for simultaneous detection of a wide range of pathogens, including bacteria, viruses, fungi, and even previously unknown or unculturable pathogens, offering a comprehensive view of the microbial composition in patient samples. We here utilized the host data and respiratory microbiome profile from a metatranscriptome test to build a ML classifier that can accurately differentiate LRTIs from non‐LRTIs.

Several studies have proven the potential of utilizing the host response to achieve precise diagnosis of complex infections. Charles R. Langelier et al. had built a model using the whole‐blood gene expression and demonstrated good accuracy in distinguishing patients with sepsis from those with non‐infectious systemic inflammatory conditions;^[^ ^24^ ^]^ their latest work also showed that the host gene expression profile from aspirate RNA‐seq could distinguish LRTIs patients from patients with non‐infectious mimics in critically ill children.^[^ ^25^ ^]^ Nevertheless, these methods require a standard RNA‐seq of clinical samples to obtain the expression levels of signatures genes and apply to the model for classification. Unlike traditional diagnostic methods that typically focus on detecting specific pathogens or require multiple tests, our approach simultaneously identifies a broad range of microorganisms, including previously unknown or unculturable pathogens, while also capturing the host's immune response. In our study, we performed transcriptome analysis of the host reads generated in the metatranscriptome test to profile host response and observed heightened inflammatory and neutrophil activation pathways in the patients with LRTIs.

Bacterial and viral pneumonia are different type of respiratory pathogens and both could lead pneumonia. Despite these differences, both types of pneumonia lead to substantial inflammation and activate a broad range of innate immune genes and pathways. Thus, we pooled both types of patients together to formed the LRTI group and established a classifier to differentiate LRTI from non‐infectious diseases. The universal activation of infection‐related genes was confirmed through PCA analysis showing that no difference in gene expression patterns between the 68 patients with bacterial infection and 43 patients with virus and/or fungi (Figure S3A, Supporting Information). We further performed a sensitivity analysis that excluded all non‐bacterial LRTI. The remaining LRTI patients exhibited similar differential gene expression patterns and pathway enrichment results, with many overlapping those observed in the full cohort (Figure S3B–D, Supporting Information).

The host gene expression levels derived from mNGS metatranscriptome data were also associated with patients’ clinical features. We found that the green module was positively associated with the count of neutrophils and white blood cells, LRTI status, and pathogen types (Figure 3A), indicating that genes in this model were probably deeply involved in the host's immune response to respiratory infections; this hypothesis was supported by the top 4 enriched pathways in this model, which related to chemokine and cytokine response after infection, as well as hepatitis B infection and natural killer cells activation (Figure 3B). Moreover, CXCR1 and IL1R1 were among the top 30 genes with high interaction with other genes in this module (Figure 3C). CXCR1 is a powerful neutrophils chemotactic factor that recruit neutrophils to lower respiratory tract in LRTIs.^[^ ^26^ ^]^ IL1R1 is receptor of the proinflammatory cytokines IL‐1 and involved in many cytokine‐induced immune and inflammatory responses after bacterial infection.^[^ ^27^ ^]^ These results indicated that gene modules clustered from mNGS data also reflect the host response after respiratory infection.

Since patients with LRTIs and non‐infectious mimics showed distinct host response, we attempted to establish a ML model using the DEGs to differentiate LRTIs from its mimics. We have selected 14 genes that achieved the best performance with 80.6% and 77.9% of accuracy in the discovery and validation cohort, respectively. This accuracy was inferior to the performance of an integrated classifier established by Eran Mick in critically ill children.^[^ ^25^ ^]^ Eran's model intergraded host LRTI probability, abundance of respiratory viruses, and dominance of pathogenic bacteria/fungi to collectively make determinations, from which the model could utilize more information to make decision, which may contribute its high accuracy. Moreover, our study involved immune‐compromised CAP patients, whose host response against LRTIs may be downgraded, resulting in lower tense infection signals compared to immune‐competent patients. However, our model utilized the host data from routine mNGS test of the BALF samples, with no need to performed DNA‐seq, making it a cost‐effective approach to assist LRTIs diagnosis without incurring additional test expense. Building on the two‐class model distinguishing LRTIs from non‐LRTIs, we further established a three‐class model that incorporated patients with indeterminate LRTI status. Although this three‐class model showed slightly lower accuracy in identifying indeterminate cases, it covered the entire cohort, representing a broader patient population relevant for clinical application. Integrating the two models could address the needs of diverse clinical situations.

Besides distinct host response, LRTIs patients showed decreased alpha diversity, evenness, and high microorganism burden in respiratory microbiome compared to patients with non‐infectious diseases, which was consistent with previous studies comparing the BALF microbiome diversity between patients in ICU and from healthy controls.^[^ ^18^ ^]^ Moreover, previous studies showed increased bacterial burden and enrichment of gut‐associated bacteria in the lung microbiome predicted poor outcomes in critically ill patients.^[^ ^19^ ^]^ Our metatranscriptome test also observed increased percentage of reads from microorganism and the enrichment of gut‐associated bacteria in the BALF from LRTI patients (Figure 5D,E). These results showed a single clinical metatranscriptome sequencing of BALF enables detailed profiling of host response and respiratory microbiome. We then added the microbiome features into the host classifier and improved the model performance on LRTIs diagnosis.

The integrated classifier we built enabled simultaneous pathogen detection while determining whether patients had LRTIs. Nevertheless, our study has several limitations. The LRTI status we relied on to build the model was based on retrospective clinical data examination, which may exist some bias in the LRTI status adjudication. Second, host response may be influenced by clinical therapy since BALF samples were not collected at the time of initial admission. Samples collected at the early stage of admission could yield more significant host response data for model training. Third, only patients met medical indication of BALF were included in the study, which may not represent the broader population of patients with LRTI. On average, BAL is performed on 23.5% of patients initially diagnosed with LRTIs at our hospital each year. Additionally, patients whose causative pathogens are easily detected by traditional methods may not require BALF mNGS testing and were therefore excluded from this study. Thus, generalizing our findings to other LRTI patients need further validation. Last, multicenter and prospective validation cohort were lacked to evaluate the model performance across different regions and in new patients, though the model showed robustness in a retrospective validation cohort enroll subsequently. Our approach requires further validation in a multicenter randomized clinical trial before hospital deployment.

In conclusion, a single metatranscriptome sequencing of BALF could concurrently profile host response and respiratory microbiome, allowing the development of a machine learning model that accurately differentiate patients with LRTIs from those with non‐infectious diseases that mimic LRTIs. This approach for LRTI diagnosis could assisted the clinician in interpreting mNGS report, adjusting anti‐infection strategy, and potentially improving clinical outcomes.

## 4 Experimental Section

### Study Design and Cohort

Patients suspected to have LRTIs were retrospectively enrolled in the Department of Respiratory and Critical Care Medicine, China‐Japan Friendship Hospital (CJFH) between October 2019 and October 2022. Hospitalized patients suspected to have common LRTIs, including community‐acquired pneumonia (CAP), community‐acquired pneumonia in immunocompromised host (CAP‐ICH), hospital‐acquired pneumonia (HAP), acute exacerbation of bronchiectasis (AEBX), acute exacerbation of chronic obstructive pulmonary disease (AECOPD), and lung abscess, were involved in this study. Patients' clinical data was collected from the electronic medical record (EMR) system of CJFH. Metatranscriptomic mNGS were ordered by clinical‐in‐charge based on the patient's clinical situation and clinician's evaluation that mNGS may assist the pathogen diagnosis; the researchers have no role on the decision to prescribe mNGS test. Patients were included if they meet the following criteria: (1) aged ≥18 years; (2) suspected of LRTIs; (2) underwent bronchoscopy and BALF were applied to mNGS. An episode of LRTIs in this study was defined as: (I) new or progressive infiltration, consolidation, ground‐glass opacity, or interstitial changes on chest radiograph; (II) recent‐onset/worsening cough with sputum production, or exacerbation of the existing respiratory symptoms, with or without phlegm, chest discomfort, dyspnea, or hemoptysis; (III) fever; (IV) signs of lung consolidation and/or auscultatory findings such as altered breath sounds and/or localized rales; (V) peripheral blood WBC > 10 ×10^9^ /L or < 4 ×10^9^ /L. If meet (I) and any of (II)–(IV), an initial diagnosis of LRTIs would be established. Typically, the medical indication for performing a BALF including: (I) The etiological diagnosis remains unclear when using other respiratory samples. (II) Patients who have inadequate response to empirical treatment and are suspected to be infected with unusual pathogens. (III) Patients without improvement after active anti‐infective therapies, who require differential diagnosis with infectious pulmonary diseases.^[^ ^28^ ^]^ BALF indications for patients in this study were summarized in Table S1 (Supporting Information). Patients were further assigned to two groups based on discharge diagnosis and clinical microbiologic findings as follows (a) Confirmed LRTIs, if the clinician made a discharge diagnosis of LRTIs and the patient had positive clinical microbiology findings; (b) non‐infectious diseases (non‐LRTIs), if clinicians made a discharge diagnosis of noninfectious disease and no respiratory pathogens detected after comprehensive clinical microbiology test. Patients who did not fall into either of the above two groups were assigned to the undetermined LRTIs group. The study was approved by the ethics committee of CJFH (2023‐KY‐302).

### Clinical Microbiology Test

Bronchoalveolar lavages were performed for all the enrolled patients during the first week after admission. All the BALF samples were applied to smear stain and culture to detect bacterial and fungal pathogens. Other assays, including PCR assays and antigen tests were performed if any pathogens were suspected. Besides BALF, sputum was also used to culture, antigen tests, and PCR if needed. Oropharyngeal swabs were applied to PCR assays, blood was applied to blood culture and cryptococcus antigen tests, and urine samples were utilized to streptococcus pneumoniae and legionella pneumophila antigen tests. The available conventional microbiologic tests performed in this study were detailed in a previous study,^[^ ^29^ ^]^ which covered common respiratory virus, bacteria, fungi, mycobacterium, mycoplasma, and chlamydia. The clinician in charge determined what microbiologic tests to perform based on the patient's clinical manifestation.

### Metatranscriptomic mNGS Test of BALF

BALF samples were transferred to three in Vitro diagnostic laboratories for RNA sequencing, namely, the BGI Genomics (Shenzheng, China), the GensKey Medicine (Tianjing, China), and the Vision Medicals (Guangzhou, China). The samples were preceded to total RNA extraction after bead‐based lysis. RNA was reverse transcribed to produce cDNA, which was used to fragmentation and adaption ligation. The RNA‐seq libraries were sequenced on Illumina Nextseq 500 with a 50 or 75 bp of length to minimize the turnaround time for pathogen detection.

### mNGS Pathogen Identification

Host reads were filtered out by aligning the total reads to human GRCh38 Genome Reference and the remaining reads were subjected to microbes’ identification using the pipeline and database covering known microbes in the NCBI database, as described in the previous study.^[^ ^30^ ^]^

### Host Gene Expression Analysis

The BALF RNA‐seq reads were quality filtered using fastp^[^ ^31^ ^]^ v0.22.0 to remove reads with a Phred score below 25 and a length under 50 bp. The filtered reads were aligned to the human reference hg38 using HISAT2 v2.2.1.^[^ ^32^ ^]^ The gene count matrix was calculated using featureCounts v1.6.3^[^ ^33^ ^]^ and normalized to counts per million reads (CPM) using edgeR v3.42.2.^[^ ^34^ ^]^ Samples with less than 300 000 estimated counts aligned were excluded from further analysis. Genes were retained for differential expression (DE) analysis if they were expressed in at least 60% of the samples. DE analysis was performed using edgeR and differentially expressed genes (DEGs) were identified with foldchange > 2 and P value < 0.05 between two groups. Heatmaps of the top 50 DEGs by absolute log2 fold changes were further generated.

Enrichment analysis of Gene Ontology and KEGG pathways was performed using the R package clusterProfiler v4.8.1^[^ ^35^ ^]^ using the DEGs at the background of all genes. Significant pathways and upstream regulators were defined as those with a gene set *p* value < 0.05.

### Weighted Gene Co‐Expression Network Analysis (WGCNA)

WGCNA analysis was performed using the R package WGCNA v1.72.^[^ ^36^ ^]^ The following clinical traits were involved in the WGCNA analysis: age, immune status, pathogen type (virus, bacteria, fungi., et al.), LRTI status (LRTI or non‐infectious illness), diagnosis regarding LRTI types, admission to ICU or not, outcome, white blood count, neophiles count, lymphocyte count, red cell count, hemoglobin, hematocrit, platelet count, alanine aminotransferase (ALT), aspartate aminotransferase (AST), creatinine, blood urea nitrogen. Missing values were imputed using the median of the available data, and the proportion of samples with missing traits was kept below 20% among each trait. High expressed gene with at least 10 counts in at least 90% of the samples were used to automatic network construction and module generation. For each module, gene significance (GS) was defined as mediated p‐value of each gene (GS = lgP) in the linear regression between gene expression and the clinical traits, which represented the association between genes and clinical traits. The module eigengene (ME) was a weighted average gene expression value and indicated the overall expression level of the module, and module membership (MM) represented the association between genes and MEs. Then, pearson's correlation analysis was performed on MEs and clinical traits, allowing the identification of the modules which were significantly associated with the external traits. Genes clustered in interested module were applied to KEGG pathway enrichment analysis and the top hub genes were visualized using Cytoscape v3.10.^[^ ^37^ ^]^

### Host Response Classifiers for LRTIs Diagnosis

To build a classifier to differentiate LRTI form its mimic non‐infectious diseases, three machine learning (ML) models were built using the scikit‐learn v1.2.0^[^ ^38^ ^]^ module on Python v3.8.2. A binary classification problem was defined, with “LRTI” patients assigned as positive class and non‐infection illness as negative class. The normalized expression level of DEGs was utilized as features input into the models. Random forest (RF), logistic regression (LR), and support vector machine (SVM) model were tested, and logistic regression was selected based on its better performance under tuned parameters (detailed in Results Section). Briefly, n_estimators, max_depth, max_features in random forest, penalty, solver, C, max_iter in the logistic regression, kernel in SVM were tuned to achieve the highest fivefold cross‐validation accuracy.

The cohort enrolled between October 2019 and October 2021 was set as discovery dataset, which was used for model training and hyper parameter tuning. The patients involved between November 2021 and December 2022 were set as validation dataset. Only DEGs identified in the discovery dataset were used as input during the model establishment stage. To improve the LR model performance, feature selection was further carried out using the “Recursive Feature Elimination ” method through the RFE class in the feature_selection module, which recursively eliminated the less important features based on their importance rankings. The maximum number of features was restricted to 20 genes to facilitate model interpretability.

After feature selection, the discovery dataset was split into training and test sets with sample size ratio of 7:3. The training dataset was used to fit the model, and its performance was evaluated on a test set. Key parameters of each model were tuned in the training sets and evaluated its impact on test set, as described previously.^[^ ^39^ ^]^ The state‐of‐art model with tuned parameter were evaluated using the validation dataset.

A fivefold cross‐validation was implemented to assess the model robustness in the discovery dataset. The dataset was split into 5 equal parts, such that in each train/test split, 4 parts were used for training and one part for testing. In the 5 rounds, every part appears in the training and testing dataset; meanwhile, in each round, a receiver operating characteristic (ROC) curve was drawn and the area under the curve (AUC) was calculated using the validation dataset and testing dataset.

Besides the two‐class model, patients with undetermined LRTIs status were included in the discovery cohort (LRTIs and non‐LRTIs) to develop a three‐category classifier, reflecting real‐world clinical scenarios in which determining infection status can be challenging. The SVM model was chosen for feature selection using the methods mentioned above for its better performance in multiple‐class differentiation. After training the classifier, the coefficients corresponding to each feature were extracted for each category. The model's performance was further assessed using fivefold cross‐validation, and the ROC curve for each category was drawn using the same method described above.

### Respiratory Microbiome Analysis

Reads passed quality control was applied to host reads removal using KneadData v0.12.0. The coverage of each microbiome and the captured diversity were evaluated using Nonpareil v3.401.^[^ ^21^ ^]^ Species‐level microbiome profiling was performed using Kraken v2.1.3 with default parameters using the standard database containing human, bacterial, fungal, archaeal, and viral genomes. The percentage of reads assigned to each taxon was then calculated. The ratio of reads from kingdom of bacteria, virus, and fungi to total reads was compared between the two cohorts. A group of five bacteria found in most healthy individuals, composed of the genera Prevotella, Streptococcus, Veillonella, Fusobacterium, and Haemophilus, has been proposed as a pulmonary core microbiota essential for lung homeostasis; the percentage of reads assigned to this lung core microbiota was then calculated for each sample. Only species with a maximum abundance exceeding 0.1% and an average abundance above 0.01% across all samples were included for further analysis. Alpha diversity was expressed as the Shannon index for normalized numbers of sequences for each sample, while evenness was expressed as Pielou index, both using the vegan package v2.6.4 in R v4.3.0. The associations between individual microbiome features and LRTI status were assessed using the general linear model, adjusting for age and antibiotic usage. Differences in beta diversity were assessed using PERMANOVA on Bray–Curtis distances as implemented in R's vegan package (Bray–Curtis distances at 1000 permutations). Beta diversity was visualized using principal coordinates analysis (PCoA) of the Bray–Curtis distances to capture the essential aspects of beta diversity. Differential abundant species between LRTI and non‐LRTI patients were identified using the Wilcoxon rank‐sum test and linear discriminant analysis (LDA) effect size (LEfSe) tools in the ImageGP web server.^[^ ^40^ ^]^ Species with p values less than 0.05 in the Wilcoxon rank‐sum test and an LDA score greater than 2 in the LEfSe analysis were selected.

### Diagnosis of LRTI Status Based on Integration of Host and Respiratory Microbiome Features

To improve the model performance on LRTIs differentiation from non‐infectious disease, microbiome features that were different between the two cohorts were integrated with host gene markers to establish an integrated classifier. Briefly, the microbiome alpha diversity (Shannon index), evenness (Pielou index), and microorganism burden (log transformed ratio of reads from the kingdoms of bacteria, fungi, and virus to total reads) were integrated into the abundance data of host marker genes to establish an integrated feature dataset. The integrated classifier was then trained on all the patients with definite LRTI and non‐infectious diseases and its performance was evaluated using fivefold cross‐validation of all the data.

## Conflict of Interest

The authors declare no conflict of interest.

## Author Contributions

X.H.Z., Y.M.W., and B.C. designed the study. X.H.Z., B.L., B.H.L., and M.W.Y. collected mNGS data and patient's clinical data. X.H.Z., J.K.Z., and Y.W.N. performed transcriptome analysis. X.H.Z. and B.L. performed the machine learning analyses and evaluation of the final machine learning classifier. All authors discussed the results and contributed critical reviews to the manuscript.

## Supporting information



Supporting Information

Supporting Information

Supporting Information

## Data Availability

The data that support the findings of this study are available in the supplementary material of this article.
