# Supplementary material for: Accurate Diagnosis of Lower Respiratory Infections Using Host Response and Respiratory Microbiome from a Single Metatranscriptome Test of Bronchoalveolar Lavage Fluid
Source: Adv Sci (Weinh). 2024 Dec 18;12(6):2405087. doi: 10.1002/advs.202405087 (PMC11809327; doi:10.1002/advs.202405087)
Supplement: Supplementary file 1 — Supporting Information [file ADVS-12-2405087-s001.pdf]

ADVANCED SCIENCE
Open Access

Supporting Information

for *Adv. Sci.*, DOI 10.1002/advs.202405087

Accurate Diagnosis of Lower Respiratory Infections Using Host Response and Respiratory Microbiome from a Single Metatranscriptome Test of Bronchoalveolar Lavage Fluid

Xiaohui Zou, Mengwei Yan, Yeming Wang, Yawen Ni, Jiankang Zhao, Binghuai Lu, Bo Liu and Bin Cao**

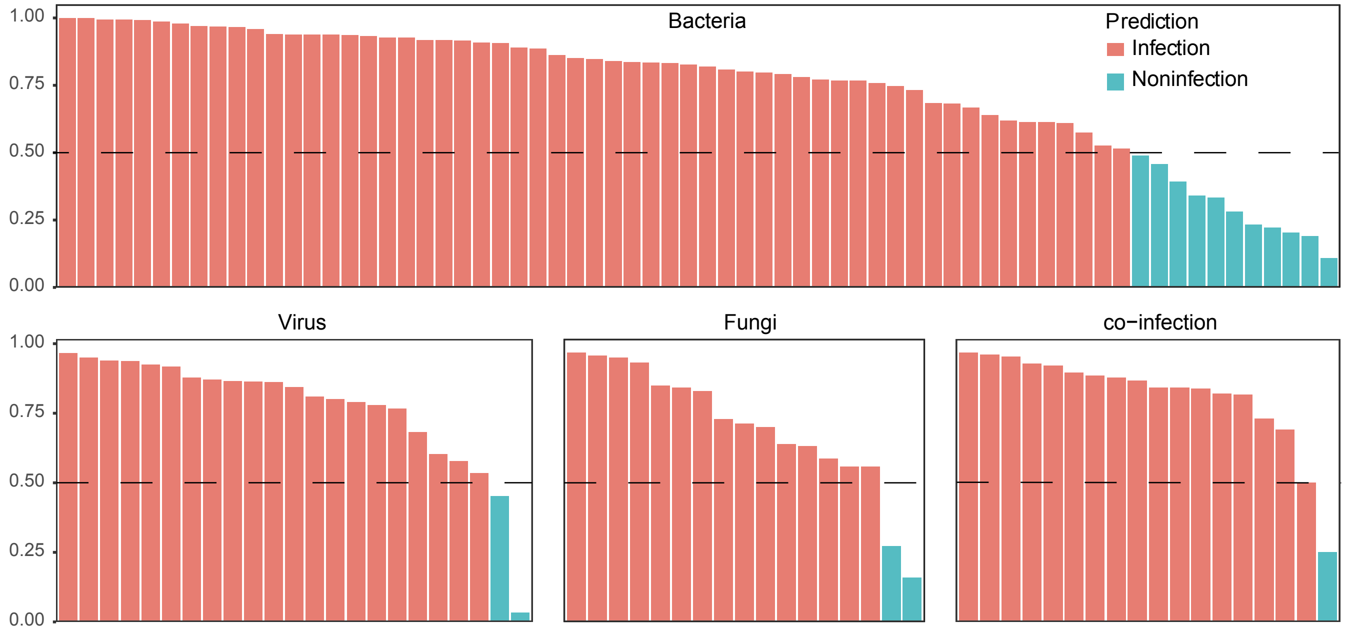

Figure S1. Performance of LRTI classifier on patients with different pathogens. The LRTI probability predicted by host-based classifier were showed for patients infected with bacteria, virus, fungi, and co-infection of different pathogens.

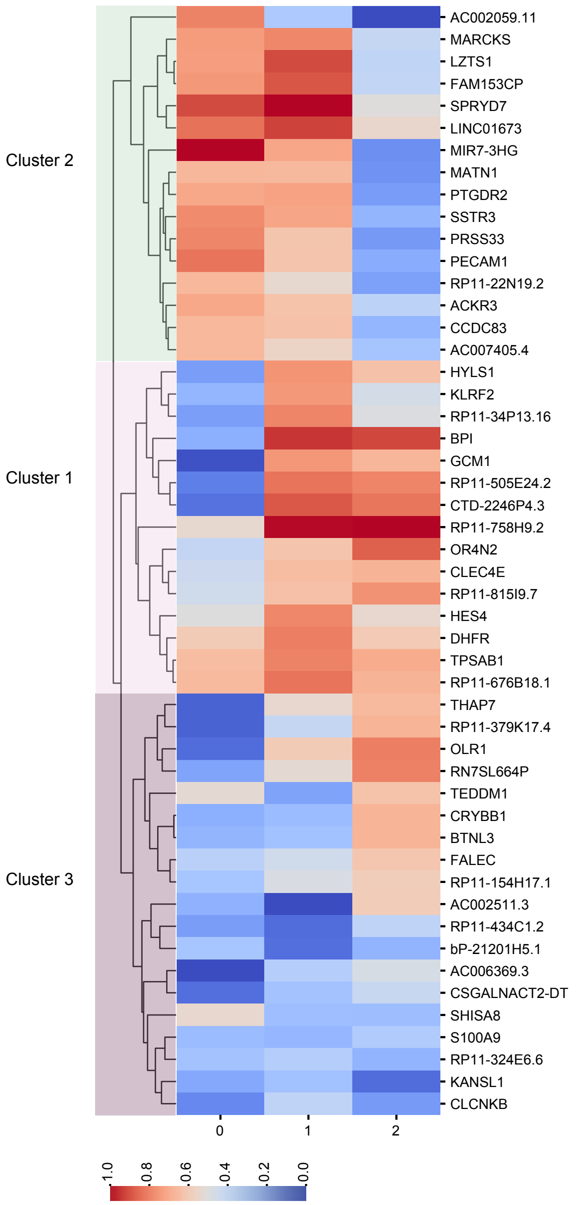

Figure S2. Coefficients of the 50 feature genes for each category in the three-class model. The colors represent the coefficients of each gene, with red indicating positive coefficients and blue indicating negative coefficients, which contribute to the classification in each category. The genes are grouped into three clusters based on their coefficients.

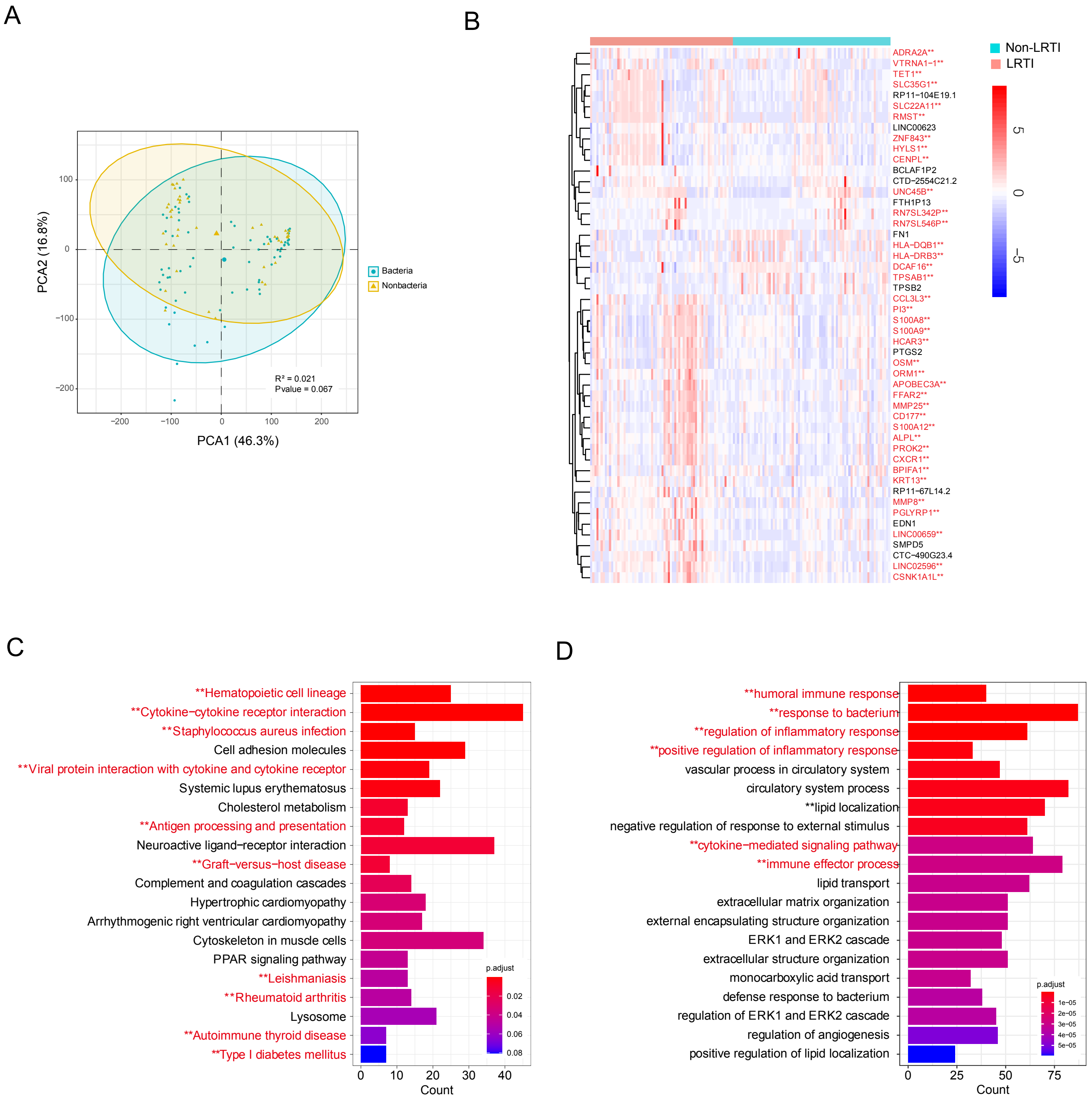

Figure S3. Sensitivity analyses of host response of LRTI patients excluding all non-bacterial patients. (A) Principal Component Analysis (PCA) plot comparing the gene expression profiles of patients with bacterial and non-bacterial LRTIs. The PCA shows no significant separation between the two groups, indicating overlapping gene expression patterns (P = 0.025, PERMANOVA). (B) Heatmap of the differentially expressed genes (DEGs) between bacterial LRTI and non-LRTI patients. (C) Enriched KEGG pathways and (D) GO terms in bacterial LRTI patients compared to non-LRTI patients. DEGs and pathways that overlap with those in the full cohort are highlighted in red and labeled with **.
